# l-Arginine Enhances Resistance against Oxidative Stress and Heat Stress in *Caenorhabditis elegans*

**DOI:** 10.3390/ijerph13100969

**Published:** 2016-09-29

**Authors:** Heran Ma, Yudan Ma, Zhixian Zhang, Ziyuan Zhao, Ran Lin, Jinming Zhu, Yi Guo, Li Xu

**Affiliations:** 1 Key laboratory for Molecular Enzymology and Engineering, The Ministry of Education, National Engineering Laboratory for AIDS Vaccine, School of Life Sciences, Jilin University, Changchun 130012, China; mahr13@mails.jlu.edu.cn (H.M.); zhangzhixian_99@126.com (Z.Z.); 18743025536@163.com (Z.Z.); avalinran@outlook.com (R.L.); 2 Sports Science Research Institute of Jilin Province, Changchun 130022, China; yudanma@sina.cn; 3 The China-Japan Union Hospital of Jilin University, Changchun 130033, China; zhujinmingWT@163.com

**Keywords:** *C. elegans*, l-Arg, antiaging, antioxidant, free radical

## Abstract

The antioxidant properties of l-arginine (l-Arg) in vivo, and its effect on enhancing resistance to oxidative stress and heat stress in *Caenorhabditis elegans* were investigated. *C. elegans*, a worm model popularly used in molecular and developmental biology, was used in the present study. Here, we report that l-Arg, at a concentration of 1 mM, prolonged *C. elegans* life by 26.98% and 37.02% under oxidative and heat stress, respectively. Further experiments indicated that the longevity-extending effects of l-Arg may be exerted by its free radical scavenging capacity and the upregulation of aging-associated gene expression in worms. This work is important in the context of numerous recent studies that concluded that environment stresses are associated with an increased population death rate.

## 1. Introduction

It is well known that the overproduction of reactive oxygen species (ROS) leads to oxidative stress. Since oxidative stress may cause cellular damage, it has been linked to several chronic diseases, including cancer, diabetes, Alzheimer’s disease, and others [1]. These diseases are characterized by an imbalance between the antioxidant enzyme system and ROS production, leading to further damage [2]. A body of evidence revealed that cellular metabolic waste products may play a major role in oxidative tissue damage [3,4]. More recently, a growing interest has been focused on the natural antioxidant compounds present in plants, animals, and even microbes [5,6,7]. As a semi-essential amino acid, l-Arg is readily available from a variety of food sources and has many important functions in living organisms as well as in various diseases [8,9,10]. Since l-Arg is used as a biosynthetic precursor of nitric oxide (NO) in mammals, it has been extensively studied and used to develop a variety of bioactive compounds. Although chronic provision of exogenous l-Arg is thought to impair NO production, known as the “arginine paradox”, acute l-Arg supplementation improves NO production and is recommended under special circumstances [11]. l-Arg concentration directly determines the activity of arginase, the production of NO, urea, citrulline and ornithine [12]. Moreover, l-Arg is also implicated in insulin resistance [13,14], heat stress [15], oxidative stress [16], and many other environmental stresses. These physiological functions of l-Arg may be attributed to its regulating effects on some aging or resistance associated genes. Liang et al. found dietary l-Arg could activate the target of rapamycin (TOR) pathway and promote TOR-related protein synthesis in *Megalobrama amblycephala* [17]. Zhu et al. reported that l-Arg supplementation could alleviate hepatic heat stress in Pekin duck [15]. The main property of l-Arg is to generate NO in vivo. Can all of these physiological effects be attributed to NO?

In this study, we used *Caenorhabditis elegans*, as a model organism to investigate whether l-Arg could enhance resistance to heat and oxidative stresses. It has been reported that NO can increase longevity, enhance stress resistance or produce other beneficial consequences [18,19,20]. Although l-Arg can be used to synthesize NO by nitric oxide synthase (NOS) for most organisms, ranging from yeasts to mammals, *C. elegans* lacks NOS and therefore cannot synthesize NO [21]. A question arises: is *C. elegans* able to enhance environmental stress resistance by directly using l-Arg, even in the absence of NO? To test this hypothesis, the worms were fed with live OP50 *Escherichia coli* as neither *C. elegans* nor *E. coli* can produce NO. These worms lived longer and were characterized by an increased activity of ROS-detoxifying enzymes and a lower production of ROS. Several factors led to longevity or increased stress resistance: some aging-associated genes or antioxidant genes were upregulated [22]. These findings are consistent with the theory that free radicals regulate the aging process.

In this article, the antiaging effects of l-Arg were investigated in vivo. l-Arg exerts a significant antioxidant activity and improves resistance to environment stresses in *C. elegans*. Our results suggest that the mechanism by which l-Arg provides strong protection against stress may be based on scavenging ROS and upregulating the expression of longevity genes, such as *hsp-16.2*, *sod-3*, *daf-2*, and *daf-16*. Since much evidence shows that environment stresses have long been associated with population death rate, this work is meaningful to further aging study.

## 2. Materials and Methods

### 2.1. Reagents

H_2_DCF-DA (2′,7′-dichlorodihydro fluoresceindiacetate), a fluorescent probe, was used to determine the ROS levels in *C. elegans*. Juglone (5-hydroxy-1,4-naphthoquinone) was used to promote ROS generation and cause oxidative stress in worms. TRIzol reagent (Invitrogen, Carlsbad, CA, USA) was used for RNA extraction. Unless otherwise stated, all reagents used were of analytical grade and obtained from Sigma Chemical Co. (Saint Louis, MO, USA).

### 2.2. Worm Strains and Maintenance

*C. elegans* was grown in a standard nematode growth medium (NGM) at 20 °C and fed with live OP50 *Escherichia coli*. Synchronization: Egg-laying worms were transferred to a new NGM plate and incubated for 3 h, following which, worms were removed and the eggs were left on the plate.

The wild-type strain Bristol N2 and the transgenic strain CF1553 (muIs84) were obtained from the Caenorhabditis Genetics Center (CGC, Sao Paulo, MN, USA). The SOD-3::GFP-linked reporter present in CF1553 was used to visualize SOD-3 expression. To visualize the expression of HSP-16.2, the transgenic strain CL2070 (dvIs70)—a gift from the University of Maryland—was employed as it contains a *HSP-16.2*::GFP-linked reporter. 

### 2.3. Stress Resistance Assay

After reaching adulthood, worms were treated with or without l-Arg (1 mM), and a sub-population was transferred into a constant temperature incubator at 35 °C. Worm death was recorded and is presented as death/h [23,24]. To detect the expression of *HSP-16.2*, worms that had just reached adulthood were heat-shocked (35 °C for 1 h), and allowed to recover for 24 h before fluorescence microscopy analysis [25].

For the oxidative stress assays, worms that had just reached adulthood were treated with or without l-Arg (1 mM) for 48 h and subsequently transferred to juglone (500 μM)-containing plates. Worm death was recorded and is presented as death/h. To detect the expression of SOD-3, 2-day-old worms were treated with or without l-Arg (1 mM) and subsequently treated with 300 μM juglone. After a 24 h recovery period, worms were observed under a fluorescence microscope.

All the aforementioned tests were repeated three times as independent parallel experiments and were conducted in a double-blind manner. All the experiments were performed with 200 worms; forty animals per group were collected from a plate well.

### 2.4. Intracellular ROS Measurement in C. elegans

H_2_DCF-DA was used as a fluorescence molecular probe to detect the concentration of intracellular ROS in worms. To increase the oxidative stress in vivo, young adult worms were pretreated with juglone (300 μM) for 1 h and subsequently cultured with or without l-Arg (1 mM) for 48 h. Afterward, worms were collected by washing the plates with M9 buffer three times to remove the remaining bacteria, and then centrifuged at low speed (4000 rpm for 30 s). Worms were then divided into a 96-well plate containing an M9 buffer. H_2_DCF-DA can penetrate into cells and bind intracellular ROS, thereby emitting fluoresce. The sample was excited at 485 nm and a microplate reader (Thermo Fisher, Waltham, MA, USA) was used collect the emission at 538 nm. Fluorescence data were collected every 30 min [26].

### 2.5. Fluorescence Quantification and Visualization

The regulatory effects of l-Arg on SOD-3 and HSP-16.2 expression were investigated. CF1553 and CL2070 worms, which just reached adulthood, were tread with or without 1 mM l-Arg. Forty worms per well (96-well plate) were suspended in a 100 μL M9 buffer and a plate reader (Thermo Fisher) was used to detect fluorescence. Five parallel experiments were performed for each group. Worms were treated as described above, were narcotized with 10 mM levamisole and fixed on a glass slide containing agarose (3%) for observation under a fluorescence microscope. The green fluorescence intensity corresponded to the expression of SOD-3 or HSP-16.2.

### 2.6. Quantitative Real-Time PCR

After 48 h of incubation with or without l-Arg (1 mM), 4-day-old worms were used for quantitative RT-PCR analysis. A TRIzol reagent was used for total RNA extraction. The primers used in RT-PCR are listed below: *ama-1*, 5′-CTGACCCAAAGAACACGGTGA-3′ and 5′-TCCAATTCGATCCGAAGAAGC-3′; *sod-3*, 5′-AGCATCATGCCACCTACGTGA-3′, and 5′-CACCACCATTGAATTTCAGCG-3′; *hsp-16.2*, 5'-CGTCGAAGAGAATACTGCTGAA-3′ and 5′-TGCAGCGAACATAACTGTATATTTAG-3′; *daf-2*, 5′-GGCCGTAGGACGTTTATTTG-3′ and 5′-TTCCACAGTGAAGAAGCCTGG-3′; *daf-16*, 5′-TTTCCGTCCCCGAACTCAA-3′, and 5′-ATTCGCCAACCCATGATGG-3′.

*Ama-1* was used as a reference gene; a Rotor-Gene 6000 RT-PCR detection system was used. RT-PCR data were analyzed using the comparative 2^−∆∆Ct^ method [27].

### 2.7. Statistical Analyses

The results were presented as mean ± standard deviation (S.D.) One-way ANOVA followed by the Tukey HSD test was performed to compare each group. Statistical significance was set at values of *p* < 0.05. All statistical analyses were conducted with Origin 8.0 software (OriginLab Co., Northampton, MA, USA).

## 3. Results and Discussion

### 3.1. l-Arg Protect Worms from Environment Stresses

**Figure 1:**
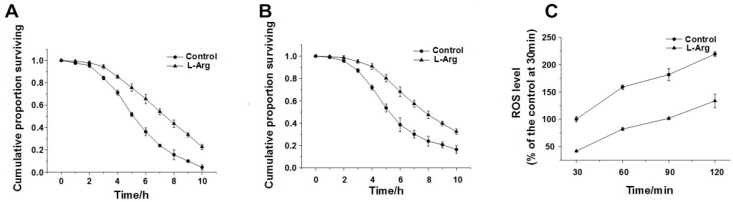
l-Arg enhances the resistance of *C. elegans* to stress conditions. (**A**) Protective effects of l-Arg (1 mM) on N2 wild-type *C. elegans* under oxidative stress; (**B**) Protective effects of l-Arg (1 mM) on N2 wild-type *C. elegans* under heat stress; (**C**) l-Arg at a concentration of 1 mM decreased ROS accumulation in *C. elegans* in response to juglone-induced oxidative stress, over time. All experiments were conducted in triplicate and were conducted in a double-blind manner.

Under stress conditions, worms activate their defense mechanisms before developing resistance. However, if worms cannot overcome the stress themselves, exogenous supplements may be useful. In oxidative stress test experiments, 2-days-old adult worms were treated with increasing l-Arg concentrations (0, 0.1, 1, 10, 50 mM) for 2 days before being transferred to a plate containing 500 μM juglone. As a pro-oxidant, juglone induces oxidative damage by converting oxygen to superoxide anion, thereby increasing intracellular ROS production [28,29]. We found that 1 mM l-Arg improved mean survival by 37.02% compared to worms under oxidative stress that did not receive amino acid supplementation (Figure 1A). Next, we examined the ability of l-Arg to protect worms from heat-stress. We found that 1 mM l-Arg (1 mM) improved the survival time by 26.98%, compared to control (Figure 1B). These results showed that l-Arg could significantly increase the mean survival time under both oxidative and heat stress. Therefore, we can hypothesize that l-Arg mixed with OP50 may be intestinally absorbed by worms and further improve resistance against environment stresses. As our result showed, l-Arg had a significant protective effect under both oxidative stress and heat stress, since the survival rate of worms corresponded with the protective ability of l-Arg on worms under environment stresses. However, chronic supplementation with l-Arg does not extend the lifespan of worms under normal conditions (Figure S1). Moreover, in order to determine whether this effect was specific to l-Arg, d-Arg was used in the same experiments and did not show any extending effects under either oxidative stress or heat stress (Figure S2A,B).

### 3.2. l-Arg Reduced the Level of Intracellular ROS

According to the free radical theory, excessive free radical accumulation harms different organisms. In the case of aerobic organisms, cell respiration is usually considered one of the chief culprits of oxidative damage as oxygen is used as an electron acceptor, hence promoting the formation and accumulation of ROS. Meanwhile, the newly generated free radicals, which mainly consist of superoxide anions, hydrogen peroxide and hydroxyl radicals, are immediately eliminated by antioxidant enzyme systems [3]. Therefore, we further analyzed whether the longevity effect of l-Arg under environmental stress was associated with its ROS scavenging ability. The usually high levels of ROS generated as a stress response were significantly decreased in worms treated with l-Arg compared to the control. We suggest that l-Arg may act as an antioxidant by scavenging free radicals accumulated in *C. elegans* and ultimately improving resistance to environment stress (Figure 1C).

### 3.3. l-Arg Upregulates SOD-3::GFP in CF1553 Worms

**Figure 2:**
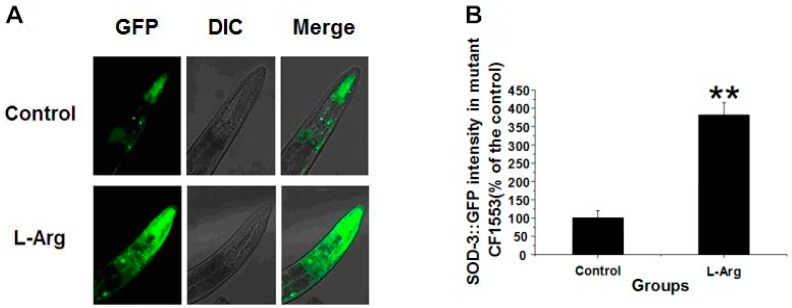
l-Arg upregulates SOD-3::GFP in CF1553 worms. (**A**) Images of SOD-3::GFP expression in l-Arg-treated and control worms; (**B**) Quantitative fluorescence analysis in transgenic CF1553 worms. The data are presented as the mean ± SE of four individual experiments, with 40 worms in each group (** *p* < 0.01).

The intracellular ROS were scavenged by l-Arg and antioxidant enzymes such as SOD-3. However, l-Arg might not only scavenge ROS, but also positively regulate the expression of antioxidant enzymes. Here, we quantitatively determine the expression of SOD-3 with a microplate reader and a fluorescence microscope. To visualize the expression of SOD-3, the transgenic worms CF1553, expressing a SOD-3::GFP reporter, were employed. l-Arg-treated worms displayed a higher fluorescence intensity than the control group (Figure 2A). When we quantified these data, we found that the expression of SOD-3::GFP was significantly upregulated by 281.12% (Figure 2B). These results suggest that l-Arg may also activate antioxidant-related signaling pathways and the consequent upregulation of SOD-3 may contribute to the increased resistance to oxidative stress. Under normal conditions, l-Arg does not show a significant upregulating effect on SOD-3 (Figure S3B,D).

### 3.4. l-Arg Upregulates HSP-16.2::GFP in CL2070 Worms

**Figure 3:**
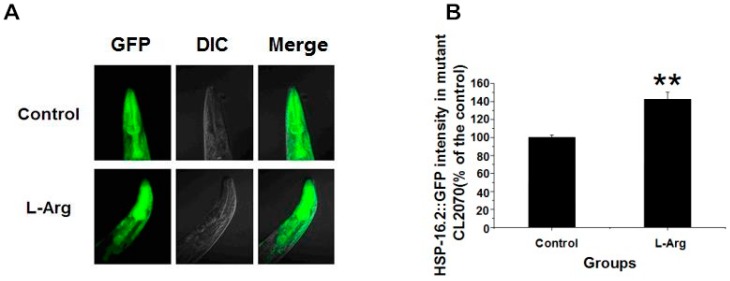
l-Arg upregulates HSP-16.2::GFP expression in CL2070 worms. (**A**) Images of HSP-16.2::GFP expression in control and l-Arg-treated worms; (**B**) Quantitative fluorescence analysis in transgenic CL2070 worms. Data are presented as the mean ± SE of four individual experiments, with 40 worms in each group (** *p* < 0.01).

HSP-16.2 is generally accepted as an indicator of longevity in *C. elegans*, and high HSP-16.2 expression is usually linked to a longer lifespan [25,30]. We therefore tested whether l-Arg might increase HSP-16.2 expression in CL2070 worms, expressing the recombinant HSP-16.2::GFP reporter gene. Worms underwent a heat-shock before a 24-h recovery period prior to fluorescence measurement. Compared to the control group, l-Arg treated worms displayed a higher degree of fluorescence (Figure 3A). When we quantified data, we found that the fluorescence intensity increased by 42.28% compared with the control (Figure 3B). Since the fluorescence intensity corresponds to the expression of HSP-16.2, we conclude that the l-Arg might prolong the meansurvival time under heat-stress by upregulating HSP-16.2. Under normal conditions, l-Arg did not show a significant upregulating effect on HSP-16.2 (Figure S3A,C).

### 3.5. l-Arg Regulates the Insulin/IGF Signaling Pathway in C. elegans

**Figure 4:**
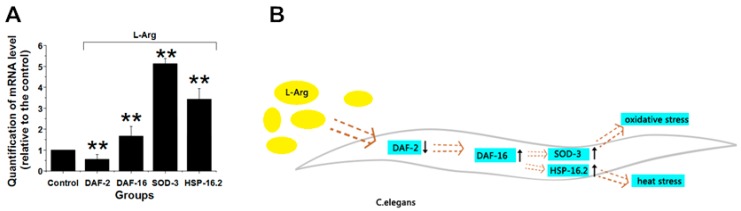
The regulatory effects of l-Arg on ageing-associated genes in *C. elegans*. (**A**) Worms were treated with or without 1 mM l-Arg. RT-PCR was performed to quantify the expression of the ageing-associated genes in *C. elegans*. Data are presented as mean ± SE (** *p* < 0.01); (**B**) Proposed mechanism of action: l-Arg regulates IIS in *C. elegans* by downregulating the expression of DAF-2 receptor and subsequently triggering the overexpression of daf-16 and its downstream target genes, and ultimately enhancing the resistance against the environment stresses.

To further investigate the relationship between the longevity and the regulatory effect of l-Arg on aging-associated signaling pathways, we performed RT-PCR analysis. According to the results, l-Arg appeared to increase the expression of *daf-16* and subsequently upregulates the *daf-16* downstream genes *hsp-16.2* and *sod-3*; while reducing *daf-2* expression (Figure 4A). *hsp-16.2* and *sod-3* upregulation is directly responsible for the longevity effect of l-Arg under heat and oxidative stress [31,32]. For most species, from yeasts to humans, the mechanisms of aging are highly conserved and linked to the insulin/IGF signaling (IIS) pathway [32,33]. In nematodes, the *daf-16* gene encodes the forehead transcription factor DAF-16 which is downstream of the IIS pathway and plays a universal role in nematode aging and stress resistance [34,35,36]. In order to perform its regulatory function in the nucleus, DAF-16 needs to inhibit the IIS pathway since IIS activation will lead to DAF-16 phosphorylation and consequent retention in the cytoplasm [36,37,38,39,40]. Once DAF-16 translocates into the nucleus, it binds to various promoters to regulate the transcription of target genes, such as MnSOD (Mn-superoxide dismutase), encoded by the *sod-3* gene in *C. elegans*, which would enhance resistance against oxidative stress [41,42]. Apart from *sod-3*, many other downstream effectors exert a regulatory effect on lifespan or stress resistance [43]. Published studies have reported that the overexpression of *hsp-16.2* also plays a role in *C. elegans* longevity [25,44,45]. Therefore, the *C. elegans* insulin/IGF receptor DAF-2 negatively regulates metabolic activity and accelerates aging. Our results showed that l-Arg significantly downregulates *daf-2* and upregulates *daf-16*. The mechanism of l-Arg-induced longevity may be as follows: DAF-2 downregulation in the cellular membrane will increase DAF-16 expression while, at the same time, reduce the IIS pathway. The latter will result in the upregulation of genes such as *hsp-16.2* and *sod-3* (Figure 4B). These findings may partially explain how l-Arg enhances resistance to environmental stress, while increasing the survival rate.

## 4. Conclusions

In conclusion, we found that l-Arg extends the lifespan of *C. elegans* under both oxidative and heat stress. l-Arg not only has free radical scavenging ability but also regulates the IIS pathway and may be co-administered for improved stress-resistance in nematodes. l-Arg exists in several health products and the above findings add to a growing body of evidence that show the significance of the insulin/IGF signaling pathway as an ageing mechanism in nematodes and potentially in humans.

## Supplementary Materials

The following are available online at www.mdpi.com/1660-4601/13/10/969/s1. Figure S1: Lifespan experiment showing the effect of l-Arg on wild type *C. elegans* N2; Figure S2: d-Arg does not enhance the resistance of *C. elegans* under stress conditions. (A) d-Arg (1 mM) does not affect the longevity of wild type *C. elegans* N2 under oxidative stress; (B) d-Arg (1 mM) does not affect the longevity of wild type *C. elegans* N2 under heat stress; Figure S3: The regulatory effect of l-Arg on HSP-16.2::GFP and SOD-3::GFP in mutant worms. (A) Images of HSP-16.2::GFP expression in l-Arg-treated and control worms; (B) Images of SOD-3::GFP expression in l-Arg-treated and control worms; (C) Quantitative fluorescence analysis in transgenic CL2070 worms; (D) Quantitative fluorescence analysis in transgenic CF1553 worms. The data are presented as the mean ± SE.

## Abbreviations

The following abbreviations are used in this manuscript:

*C. elegans*	*Caenorhabditis elegans*
ROS	reactive oxygen species
l-Arg	l-arginine
d-Arg	d-arginine
MnSOD	Mn-superoxide dismutase
IIS	insulin/IGF signaling
