# Supplementary material for: l-Arginine Enhances Resistance against Oxidative Stress and Heat Stress in Caenorhabditis elegans
Source: Int J Environ Res Public Health. 2016 Sep 29;13(10):969. doi: 10.3390/ijerph13100969 (PMC5086708; doi:10.3390/ijerph13100969)
Supplement: Supplementary file 1 [file ijerph-13-00969-s001.pdf]

Supplementary Materials: L-Arginine Enhances Resistance against Oxidative Stress and Heat Stress in *Caenorhabditis elegans*

Heran Ma, Yudan Ma, Zhixian Zhang, Ziyuan Zhao, Ran Lin, Jinming Zhu, Yi Guo and Li Xu

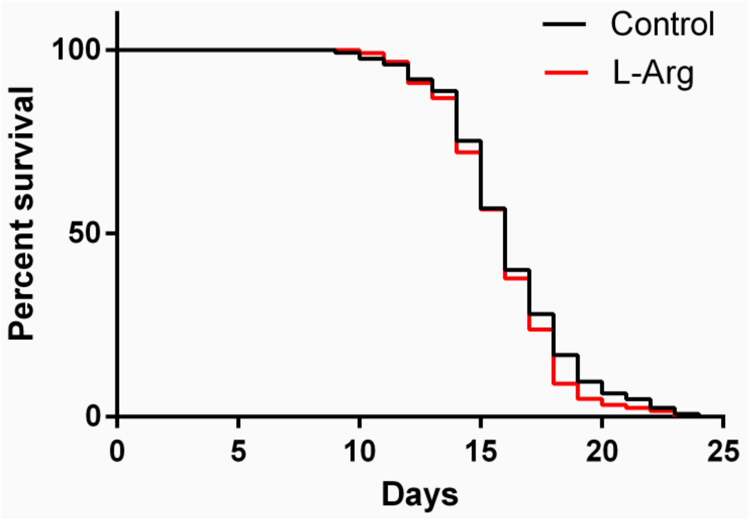

Figure S1. Lifespan experiment showing the effect of L-Arg on wild type *C. elegans* N2.

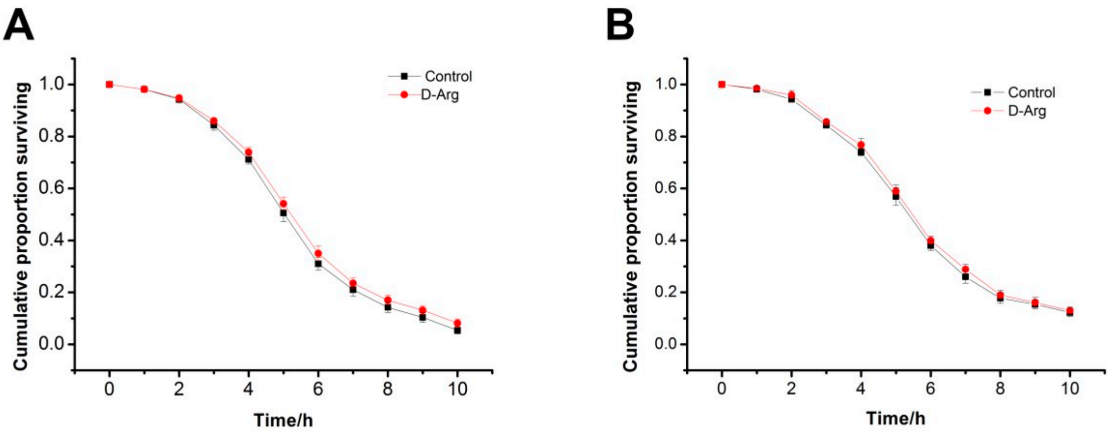

Figure S2. D-Arg does not enhance the resistance of *C. elegans* under stress conditions. (**A**) D-Arg (1 mM) does not affect the longevity of wild type *C. elegans* N2 under oxidative stress; (**B**) D-Arg (1 mM) does not affect the longevity of wild type *C. elegans* N2 under heat stress.

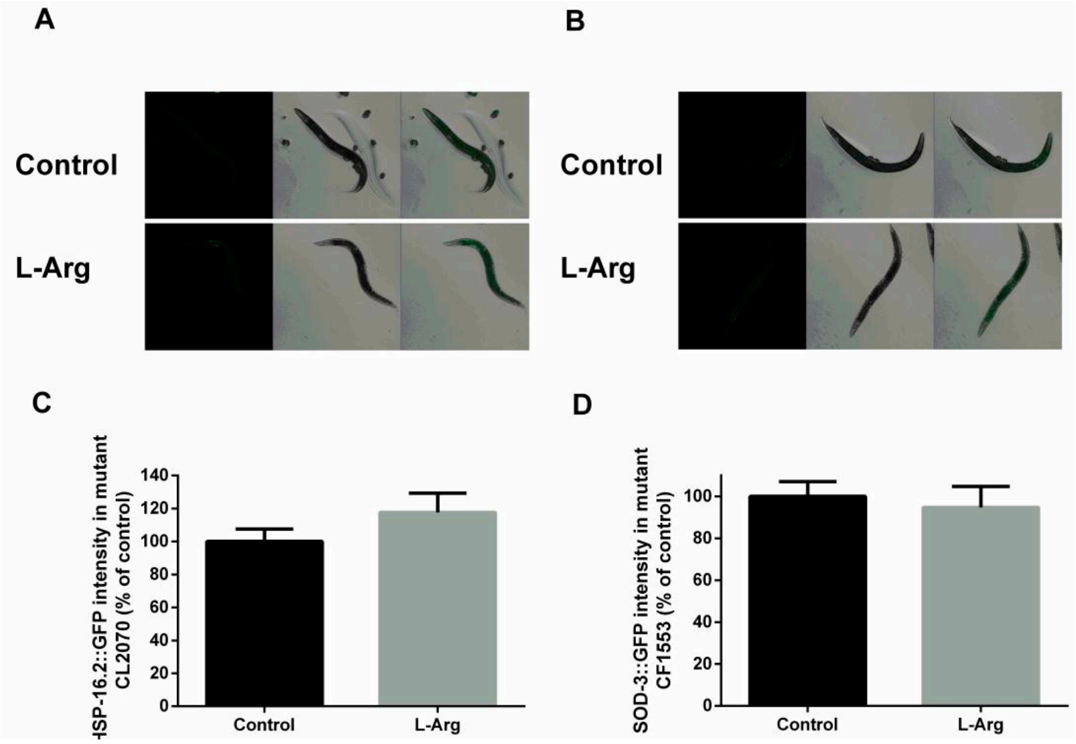

Figure S3. The regulatory effect of L-Arg on HSP-16.2::GFP and SOD-3::GFP in mutant worms. (**A**) Images of HSP-16.2::GFP expression in L-Arg-treated and control worms; (**B**) Images of SOD-3::GFP expression in L-Arg-treated and control worms; (**C**) Quantitative fluorescence analysis in transgenic CL2070 worms; (**D**) Quantitative fluorescence analysis in transgenic CF1553 worms. The data are presented as the mean ± SE.

© 2016 by the authors. Submitted for possible open access publication under the terms and conditions of the Creative Commons Attribution (CC-BY) license (http://creativecommons.org/licenses/by/4.0/).
